# Radiomics model based on multi-sequence MRI for preoperative prediction of ki-67 expression levels in early endometrial cancer

**DOI:** 10.1038/s41598-023-49540-0

**Published:** 2023-12-12

**Authors:** Si-Xuan Ding, Yu-Feng Sun, Huan Meng, Jia-Ning Wang, Lin-Yan Xue, Bu-Lang Gao, Xiao-Ping Yin

**Affiliations:** 1 Department of Radiology, Affiliated Hospital of Hebei University, Hebei Key Laboratory of Precise Imaging of Inflammation Related Tumors, No. 212 Eastern Yuhua Road, Baoding City, 071000 Hebei Province People’s Republic of China; 2 College of Quality and Technical Supervision, Hebei University, No. 180, Wu Si East Road, Baoding City, 071000 Hebei Province People’s Republic of China

**Keywords:** Cancer, Computational biology and bioinformatics, Diseases, Medical research, Oncology

## Abstract

To validate a radiomics model based on multi-sequence magnetic resonance imaging (MRI) in predicting the ki-67 expression levels in early-stage endometrial cancer, 131 patients with early endometrial cancer who had undergone pathological examination and preoperative MRI scan were retrospectively enrolled and divided into two groups based on the ki-67 expression levels. The radiomics features were extracted from the T2 weighted imaging (T2WI), dynamic contrast enhanced T1 weighted imaging (DCE-T1WI), and apparent diffusion coefficient (ADC) map and screened using the Pearson correlation coefficients (PCC). A multi-layer perceptual machine and fivefold cross-validation were used to construct the radiomics model. The receiver operating characteristic (ROC) curves analysis, calibration curves, and decision curve analysis (DCA) were used to assess the models. The combined multi-sequence radiomics model of T2WI, DCE-T1WI, and ADC map showed better discriminatory powers than those using only one sequence. The combined radiomics models with multi-sequence fusions achieved the highest area under the ROC curve (AUC). The AUC value of the validation set was 0.852, with an accuracy of 0.827, sensitivity of 0.844, specificity of 0.773, and precision of 0.799. In conclusion, the combined multi-sequence MRI based radiomics model enables preoperative noninvasive prediction of the ki-67 expression levels in early endometrial cancer. This provides an objective imaging basis for clinical diagnosis and treatment.

## Introduction

Endometrial cancer (EC) is a common gynecological malignancy. With an increasing prevalence of obesity and continuing declines in fertility, the incidence and mortality of EC continue to rise globally^1–3^, which necessitates an urgent improvement in the diagnosis and treatment levels of EC. In addition to the traditional depth of muscular invasion, International Federation of Gynecology and Obstetrics (FIGO) staging, histologic type, and pathological grade, the expression of ki-67 can also be used to predict the recurrence and prognosis of endometrial cancer^4,5^. Ki-67 is a proliferation marker protein and can effectively indicate the cell proliferation status and biological behavior as an important diagnostic marker in the pathological differentiation of various lesions^6,7^. The ki-67 expression can be detected by immunohistochemistry, however, immunohistochemical methods require invasive pathological biopsy of the tumor and cannot reflect the full range of ki-67 expression because of the influence of sampling size and location.

Radiomics, which combines quantitative analysis of images with machine learning methods, is an emerging approach in imaging analysis and enables analysis of intra-tumor heterogeneity. In current oncology research, radiomics is mainly applied to histopathological grading, differential diagnosis, genomic classification, survival prediction, and evaluation of treatment response^8,9^. It improves the accuracy of diagnosis and treatment response assessment, avoiding invasive medical procedures.

Current radiomics studies of EC have focused on the prediction of histological grade, depth of myometrial invasion and lympho-vascular space invasion in EC^10–18^. Most of these studies employed traditional machine learning methods. Recently, a study using deep learning methods to diagnose endometrial hyperplasia and screen for endometrial intraepithelial neoplasia in histopathological images was presented^19^. The study reported that good accuracy can be achieved based on deep learning, indicating possible application in clinical practice^19^.

There are few studies using radiomics-based methods to predict ki-67 expression levels in early-stage EC. Only one study^20^ had investigated the feasibility of apparent diffusion coefficient (ADC) value combined with texture analysis in preoperatively predicting the expression levels of Ki-67 and p53 in EC patients with the logistic regression approach to construct a radiomics model, including patients with EC at all stages. The radiomics features may be different in advanced- versus early-staged EC, and the resultant radiomics model in the above study^20^ may not accurately reflect the real expression of ki-67 in early-staged EC. Therefore, in this study, we attempted to use artificial neural network methods to develop and validate a more clinically applicable radiomics machine learning model based on the multi-sequence MRI in predicting noninvasively preoperatively the ki-67 expression levels in early-stage EC so as to improve the diagnosis and treatment effect.

## Materials and methods

### Patients

This retrospective study was approved by the ethics committee of Hebei University Hospital, and all patients had given written informed consent to participate. All methods were performed in accordance with the relevant guidelines and regulations. A total of 149 female patients aged 28–74 (mean 56.5 ± 8.8) years at diagnosis of early-stage EC from January 2020 to September 2022 were retrospectively screened. Early-stage EC referred to the EC with FIGO stage I confined to the corpus uteri. The inclusion criteria were patients with primary early-stage EC confirmed by surgical pathology, pathologic immunohistochemical indices including ki-67, pelvic cavity MRI within a month before gynecological surgery, and no tumor related treatment before MRI. The exclusion criteria were absence of imaging data of T2WI, dynamic contrast-enhanced T1 weighted imaging (DCE-T1WI), or ADC map (n = 12), the largest diameter of the lesion area less than 1 cm on MRI (n = 3), and poor image quality (n = 3).

**Figure 1:**
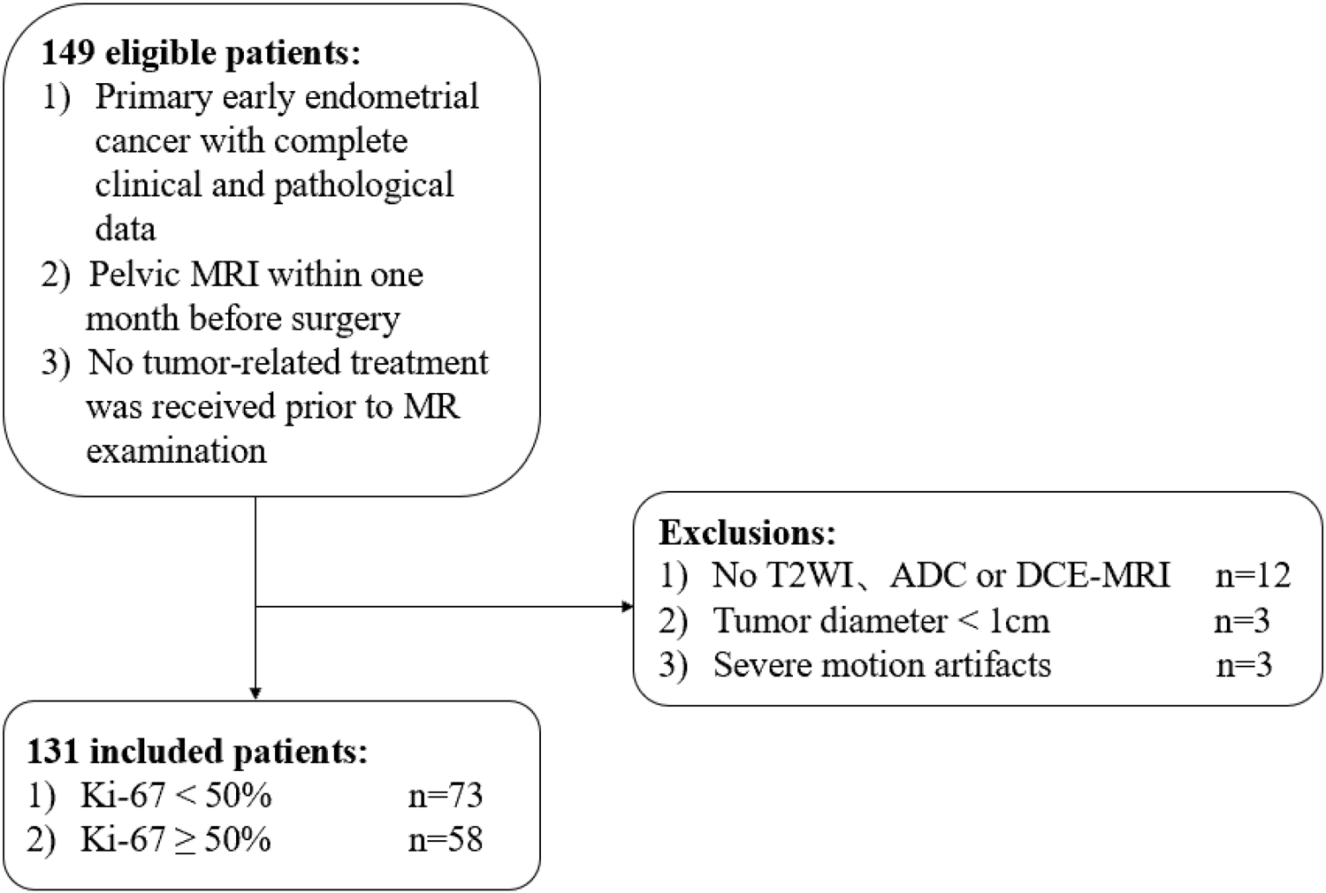
Flow chart of patient screening. T2WI, T2 weighted MRI imaging; ADC, apparent diffusion coefficient; DCE, dynamic contrast enhancement.

Ultimately, 131 patients with MRI examinations were enrolled and divided into 2 groups according to the ki-67 expression levels: low expression (ki-67 < 50%) group (n = 73) and high expression (ki-67 ≥ 50%) group (n = 58) (Fig. 1).

### Measurement of ki-67

All surgical resection specimens were sent to the pathology department for fixation, dehydration, waxing, embedding, sectioning, and staining of tissue for immunohistochemistry within one week. Detection of the ki-67 expression was performed by two professional pathologists with over ten years of experience in pathological diagnosis. The ki-67 score was obtained by recording the percentage of positively stained tumor cells. The ki-67 expression was positive when there were clear brown yellow granules within the nuclei of tumor cells. Ten fields were randomly selected under high magnification in the hot spot area, and the percentage of tumor positivity in each field was taken as the proliferation index. The patients were divided into the ki-67 ≥ 50% group and the ki-67 < 50% group according to the ki-67 value-added index^21^.

### MRI protocol

All patients underwent routine MRI examinations with a 3.0T MRI scanner (GE discovery MR 750) and a 1.5T MRI scanner (SIEMENS MAGNETOM Amira) equipped with an 8-channel torso body phased array coil. Patients were fasted for at least four hours and had their bladders moderately filled prior to scanning. The scan sequences were 1) sagittal T2WI with the following parameters of TR/TE 5800 ms/85 ms (Amira: 3160 ms/86 ms), slice thickness/slice distance 5 mm/1 mm (Amira: 4 mm/1 mm), NEX 2, field of view (FOV) 26 × 26 cm, and matrix 320 × 320, 2) DWI sequence with the parameters of TR/TE 3000 ms/minimum (Amira: 3850 ms/61 ms), slice thickness/slice interval 5 mm/1 mm (Amira: 4 mm/l mm), NEX 2, FOV 30 × 30 cm, matrix 128 × 130, b = 0, and 800 s/mm^2^, and 3) dynamic contrastenhanced scanning with the contrast agent of gadobenate-dimeglumine. The injection was administered by a median cubital intravenous bolus at a rate of 2 ml/s after the dose was calculated at 0.2 ml/kg. Images were acquired during multiple phases following administration of intravenous gadobenate-dimeglumine in either sagittal or axial oblique plane (before and at 27, 54, 81, 108, 135 and 162 s after contrast material administration in the sagittal plane and 189 s after contrast material administration in the axial oblique plane). The ADC map is automatically calculated based on DWI by the embedded software of the MRI.

### Image analysis

**Figure 2:**
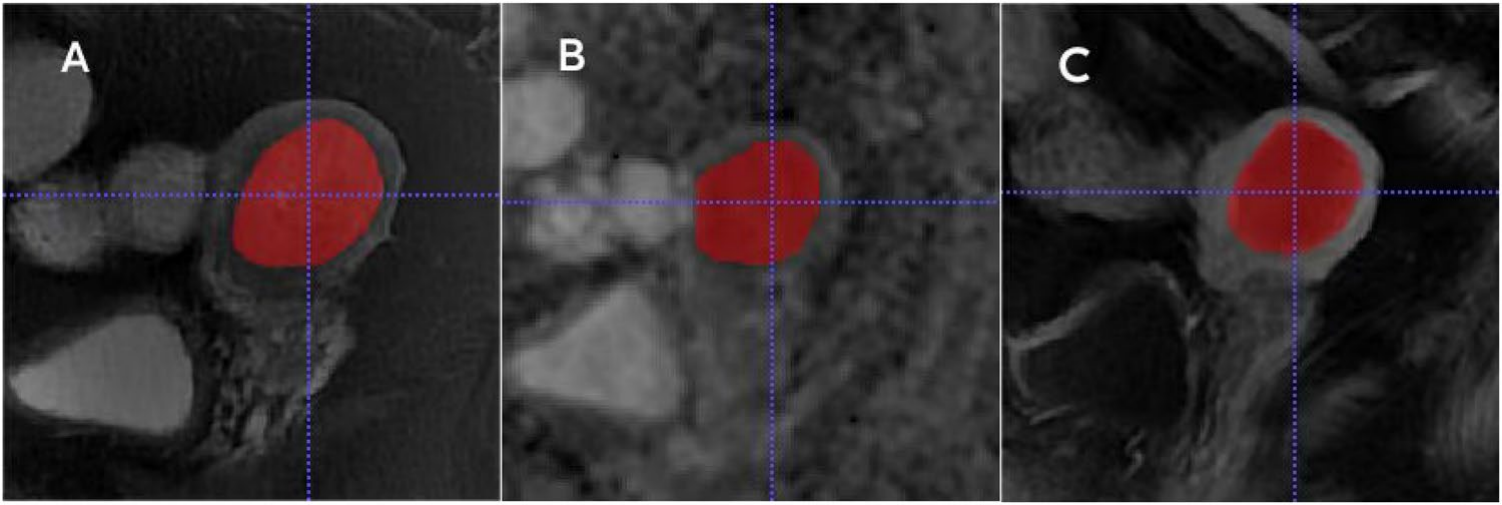
Schematic diagram of tumor segmentation. (**A**) sagittal T2WI, (**B**) sagittal ADC map, (**C**) sagittal DCE-T1WI. ADC, apparent diffusion coefficient; DCE, dynamic contrast enhanced.

The MRI raw images of all patients were exported and stored in the DICOM format before being imported into the ITK-SNAP 3.8 software for analysis. A physician with over 5 years of experience in pelvic MRI imaging diagnosis manually sketched the entire tumor in a three-dimensional region of interest (3D ROI) layer by layer on T2WI, delayed phase DCE-T1WI, and ADC image sequences. The delayed phase in this article referred to the image obtained at the sagittal position 162 s after contrast injection. All contours were reviewed by a radiologist chief physician with 20 years of experience in diagnostic pelvic MRI imaging. Any questions arose during segmentation were resolved via discussion; consensus was obtained through agreement between two radiologists. Figure 2 shows the segmentation.

Firstly, the pyradiomics toolbox 3.7 was used to extract features from the regions of interest in three sequences. In total, 1521 features were extracted, including 304 first-order features, 17 shape (in 2D and 3D) features, 384 Gy level co-occurrence matrix (GLCM), 224 Gy level dependence matrix (GLDM), 256 Gy-level run-length matrix (GLRLM), 256 Gy level size zone matrix (GLSZM), and 80 neighboring gray tone difference matrix (NGTDM).

Secondly, the Pearson correlation coefficient (PCC) was used for screening of radiomics features and removal of features with high redundancy and low correlation. The data were then normalized using a Z-score, which avoided excessive network prediction error due to the large orders of magnitude difference in input–output data. After that, the data were augmented using the borderline smote method to solve the problem of imbalance in the sample size of the study subjects.

**Figure 3:**
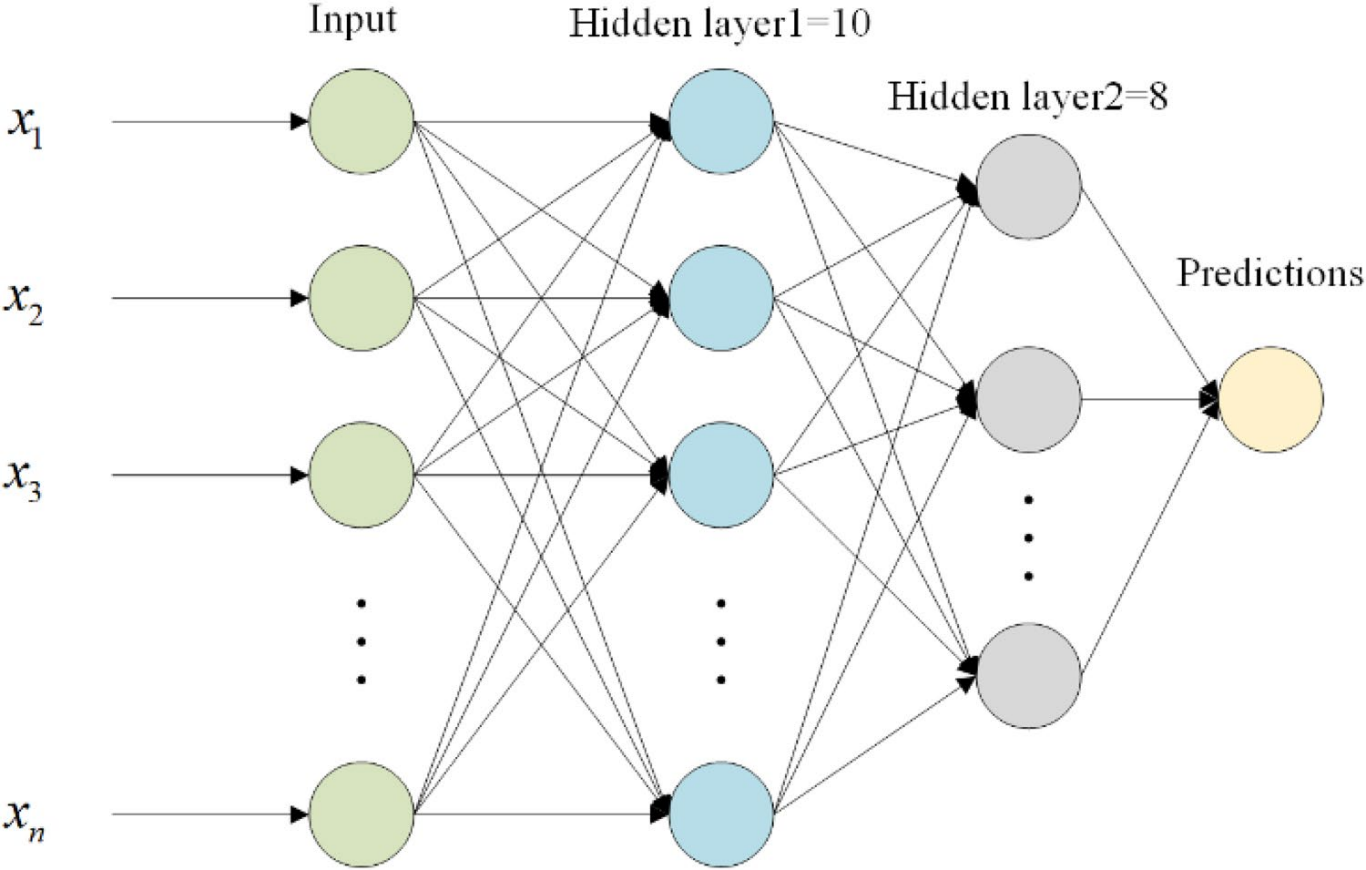
The MLP structure maps built with a PyTorch framework. MLP, multilayer perceptual machine.

**Figure 4:**
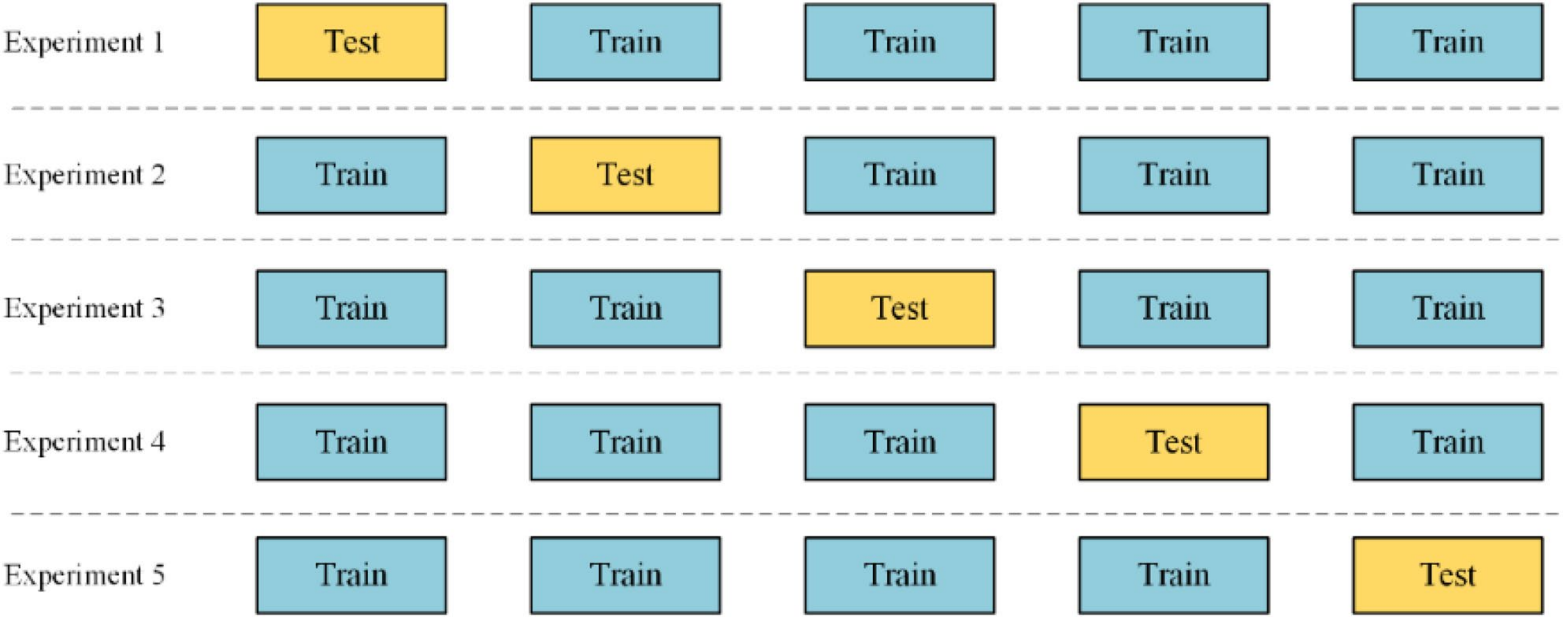
Data partitioning diagram for the five-fold cross validation.

Finally, the radiomics features were trained by a multilayer perceptual machine (MLP) built with a PyTorch framework (PyTorch 1.13). The features were validated in a five-fold cross validation. The ratio of the training set to the test set was 4:1, and the final experimental results were averaged from the results of five validations. Figure 3 presents the MLP structure maps, and Fig. 4 demonstrates the five-fold cross validated data partitioning method.

### Statistical analysis

The Python3.7, SPSS 22.0 and MedCalc19.04 software were used for statistical analysis. Independent samples t-test, Chi square test and Mann Whitney U test were used to compare the differences in imaging characteristics and clinicopathological characteristics between the ki-67 ≥ 50% and ki-67 < 50% groups. The DeLong’s test was used to analyze the improvement resulting from the combined model compared to the other single models. *P* < 0.05 was considered statistically significant. The extracted radiomics features were screened by PCC. The area under the curve (AUC) of the receiver operating characteristics (ROC) analysis, sensitivity, specificity, accuracy, and precision were used for model performance evaluation. Calibration curves were used to assess the goodness of fit of the models. Decision curve analysis (DCA) was conducted to estimate the clinical usefulness of the models by calculating the net benefits at different values of threshold probability.

## Results

### Patient characteristics and surgical histopathologic findings

A total of 131 patients were enrolled, including 73 in the ki-67 < 50% group and 58 in the ki-67 ≥ 50% group (Table 1). A significant (*P* < 0.05) difference existed in the tissue differentiation and histology type between the ki-67 < 50% and the ki-67 ≥ 50% groups.

**Table 1:** Patients clinical and pathological characteristics.

Variable	ki-67 < 50% (n = 73)	ki-67 ≥ 50% (n = 58)	*P*-value
Age	57.38 ± 8.84	56.93 ± 7.90	0.342
FIGO stage			0.749
IA	51	42	
IB	22	16	
Differentiation			0.000
G1	42	18	
G2	26	18	
G3	5	22	
Histology			0.006
Type I	70	47	
Type II	3	11	

*p < 0.05.

### Radiomics signature analysis

**Figure 5:**
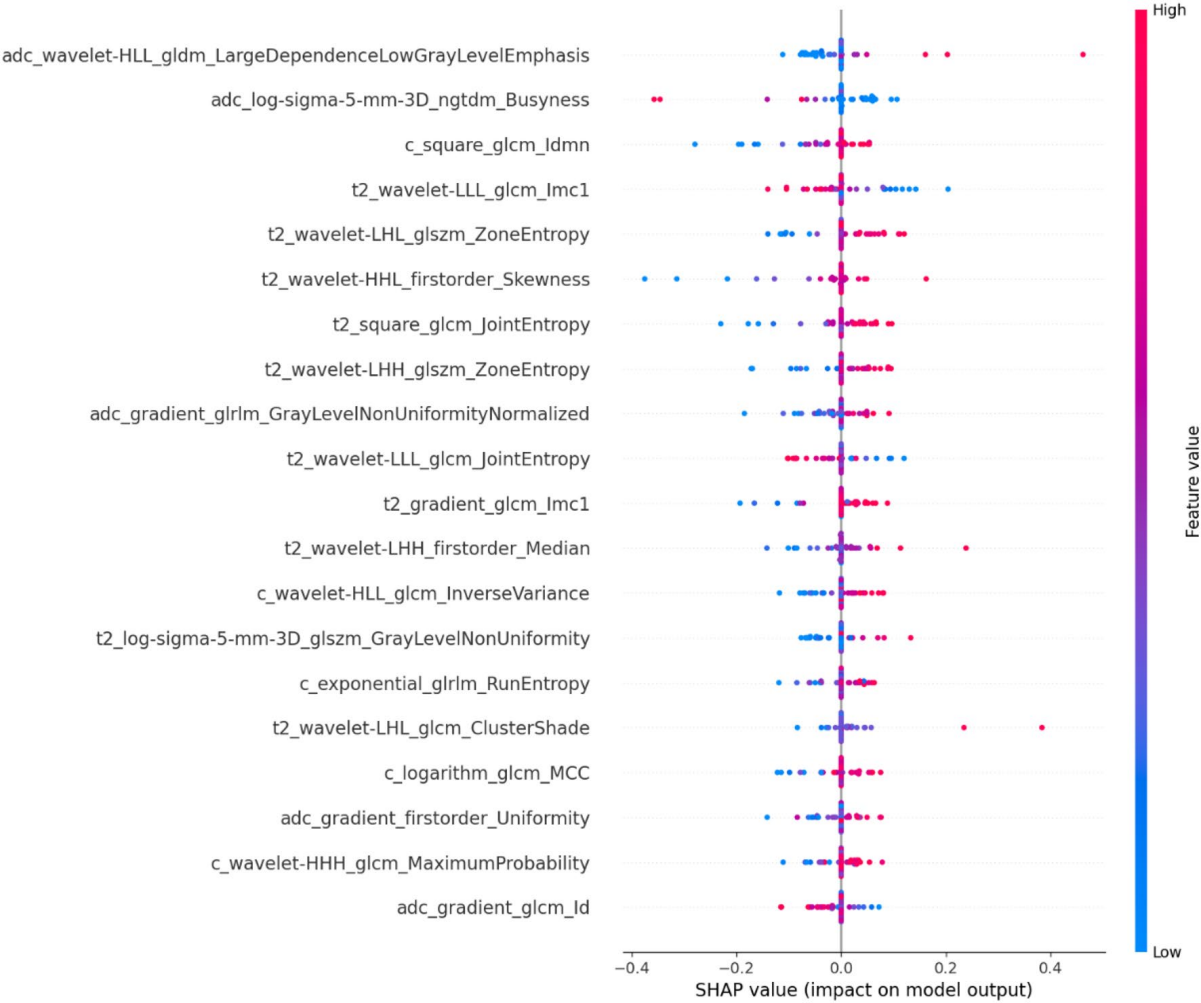
The shape plot of the top 20 features.

A total of 1521 features were extracted from the MRI T2WI, DCE-T1WI, and ADC maps, and PCC was used to dimensionally reduce the extracted radiomics features. Finally, 25, 31, and 20 radiomics features that were most valuable for the ki-67 classification were selected from the T2WI, DCE-T1WI, and ADC maps, respectively. Figure 5 presents a SNAP summary plot of the top 20 features showing the importance of the features and their effect on the model, each point in the plot represents the SNAP value of the sample, the y-axis represented the multimodal features sorted by importance, the x-axis represented the size of the SNAP value, and the color represented the size of the feature value. Among the top twenty features which had the highest number of features extracted from T2WI in the SNAP maps, the GLDM features of the ADC maps were most important for the ki-67 prediction in EC.

### Construction and validation of the radiomics model

**Figure 6:**
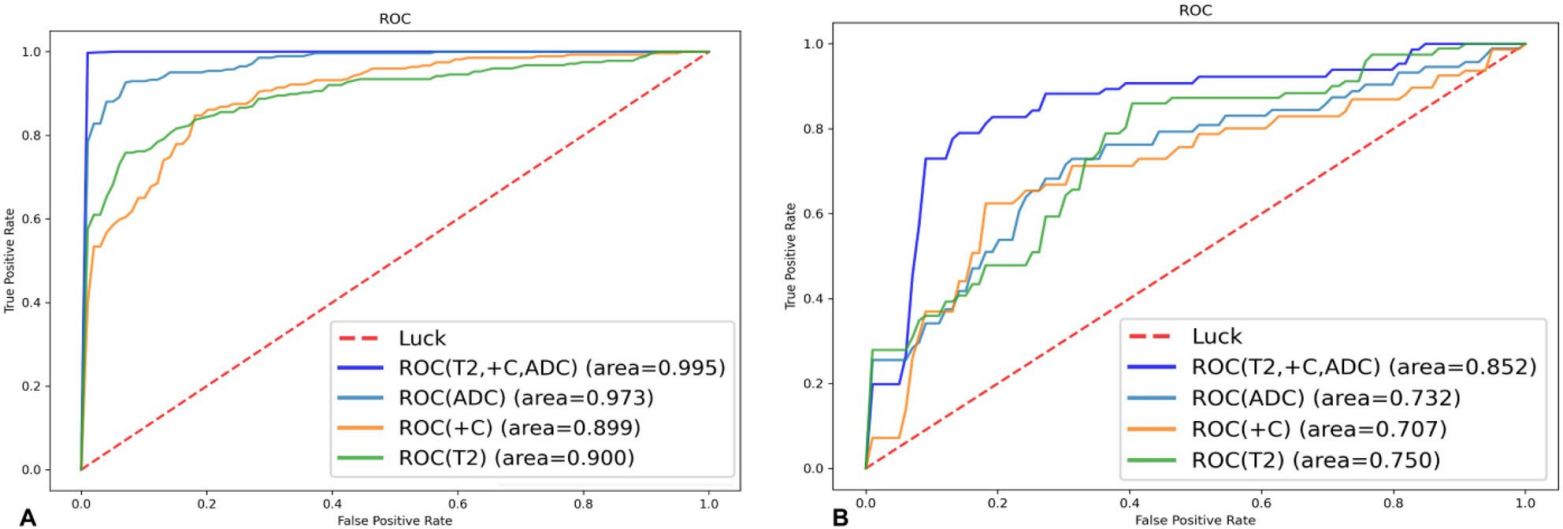
Receiver operating characteristics (ROC) curve analysis for prediction of the ki-67 expression by the MLP model. Equality of AUC was assessed by the DeLong’s test. (**A**) Training set. (**B**) Validation set.

**Figure 7:**
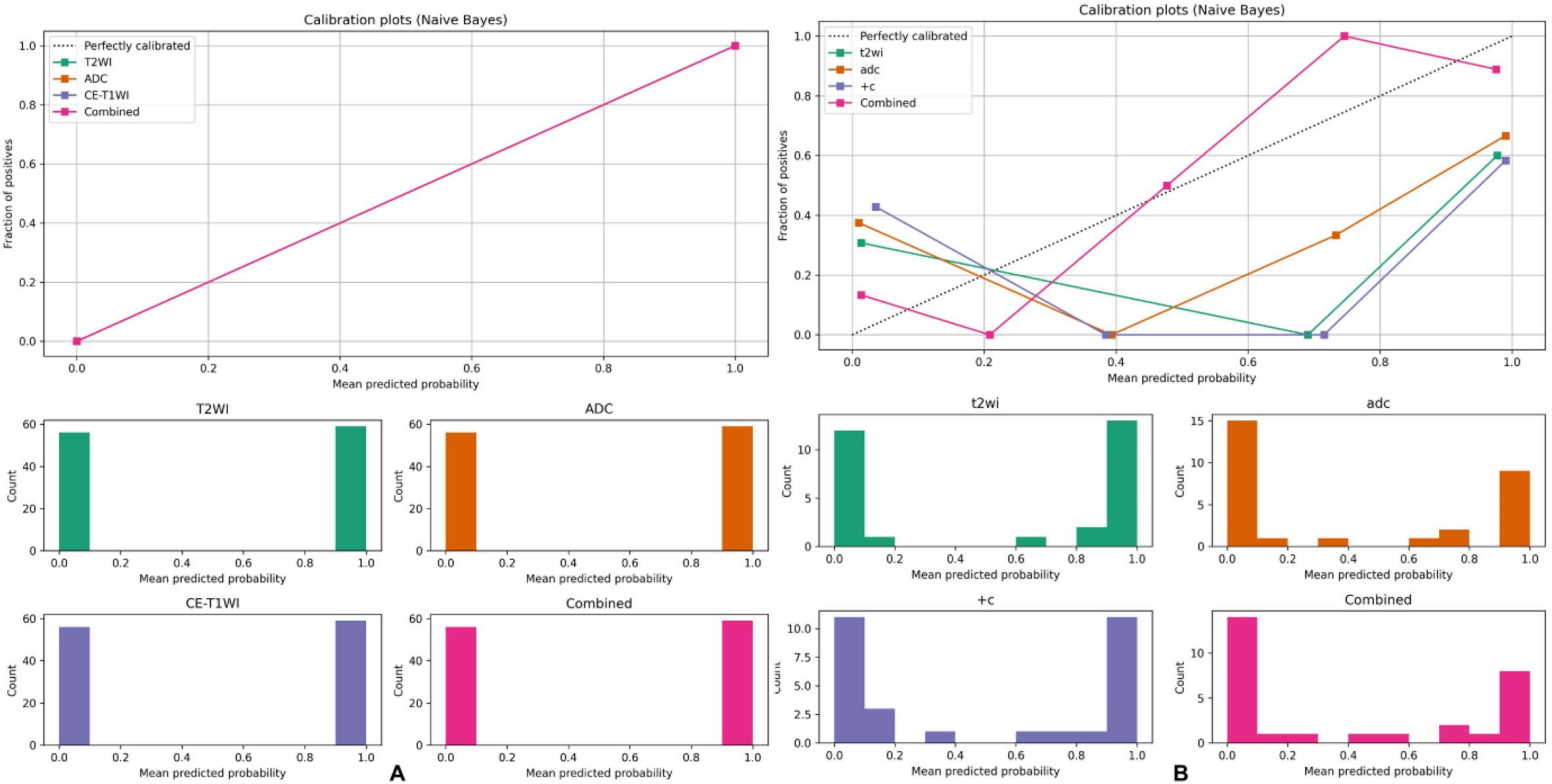
Calibration plots of the four models in the training and validation set. (**A**) The calibration plots of the four models in the training set. (**B**) The calibration plots of the four models in the validation set.

**Figure 8:**
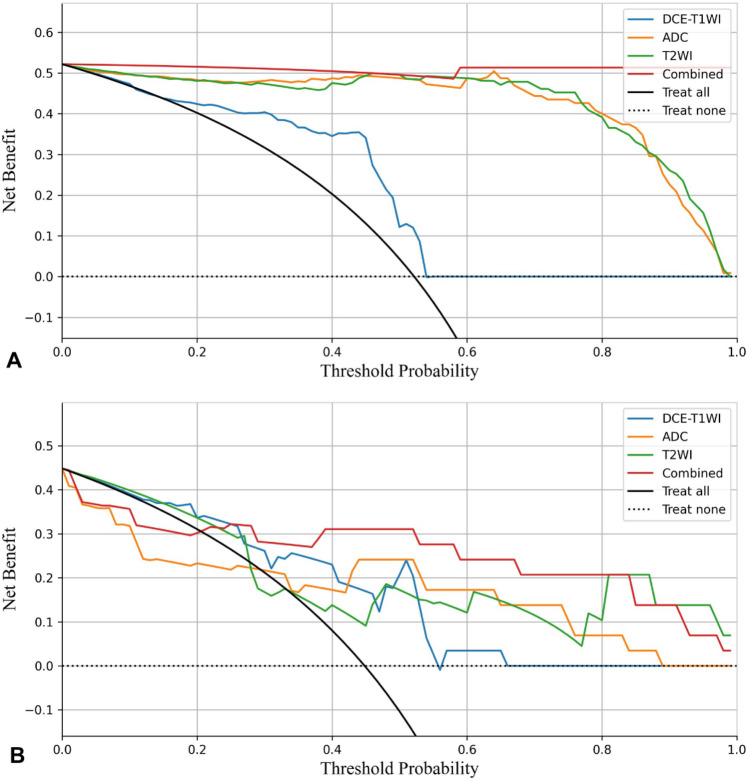
Decision curve analysis of four models in the training and validation sets. (**A**) Decision curve analysis of four models in the training set. (**B**) Decision curve analysis of four models in the validation set.

Table 2 shows the AUC, accuracy, sensitivity, specificity, and precision of the MRI-based MLP model for predicting the ki-67 expression levels. The potency of the MLP model for ki-67 prediction based on single and combined sequences was assessed using the ROC curve analysis (Fig. 6). The DeLong’s test was used to analyze the improvement resulting from the combined model compared to the other single models (Table 3), with the model based on the combined T2WI, DCE-T1WI, and ADC images showing the best efficacy. The AUC value for predicting the ki-67 expression level in the validation set was 0.852, with an accuracy of 0.827, a sensitivity of 0.844, a specificity of 0.773, and a precision of 0.799. Calibration curves showed good fitness for the combined model (Fig. 7). DCA of the models is shown in Fig. 8.

**Table 2:** Performances of different models in the validation sets.

Model	Date set	AUC (95%CI)	Accuracy	Precision	Sensitivity	Specificity
T2WI	Training set	0.900 (0.786–1.000)	0.846	0.860	0.837	0.854
	Validation set	0.750 (0.666–0.834)	0.736	0.767	0.699	0.782
ADC	Training set	0.973 (0.942–1.000)	0.932	0.939	0.927	0.936
	Validation set	0.732 (0.721–0.743)	0.750	9.736	0.790	0.714
DCE-T1WI	Training set	0.899 (0.798–1.000)	0.791	0.790	0.845	0.742
	Validation set	0.707 (0.642–0.771)	0.750	0.712	0.839	0.634
Combined	Training set	0.995 (0.992–1.000)	0.993	0.997	0.989	0.997
	Validation set	0.852 (0.816–0.889)	0.827	0.799	0.844	0.773

Receiver operating characteristics (ROC) curve analysis. *AUC* area under the ROC curve; *T2WI* T2 weighted MRI imaging; *T1WI* T1 weighted MRI imaging; *DCE* dynamic contrast enhancement; *ADC* apparent diffusion coefficient; *CI* confidence interval.

**Table 3:** AUC of the four models was compared.

Model	Training set		Validation set	
	*P*-value	Z-score	*P*-value	Z-score
Combined vs T2WI	< 0.001	5.824	0.002	3.166
Combined vs ADC	0.013	1.504	< 0.001	4.623
Combined vs DCE-T1WI	< 0.001	5.102	< 0.001	3.917
T2WI vs ADC	< 0.001	−4.389	0.593	0.534
T2WI vs DCE-T1WI	0.961	0.049	0.211	1.251
ADC vs DCE-T1WI	< 0.001	3.878	0.447	0.760

*p < 0.05.

## Discussions

EC is prone to early detection because of its pathogenetic characteristics. Most patients are often in stage I at the time of diagnosis, and effective treatment is usually available at this stage. However, some early-stage patients will experience local recurrence and metastasis after treatment. Tumor markers are beneficial for identifying high-risk tumors, in which the expression level of ki-67 protein is closely related to cell proliferation. The ki-67 protein is present in all active phases of the cell cycle (G1, S, G2 and mitosis) but absent in quiescent cells (G0). This fact makes the ki-67 proliferation index a good marker for this disease during the development course. Investigators can use ki-67 to predict tumor recurrence and distant metastasis^22^. Based on this, this study proposed a multi-sequence MRI based radiomics model to preoperatively predict the ki-67 expression levels in early-stage EC.

This study found significant differences in the pathological grade and histological type between different Ki-67 groups, suggesting that patients with non-endometrioid carcinoma or pathological grade with poorly differentiated prognosis had a poor prognosis, and long-term observation or intervention was required after treatment.

The combination of T2WI, DWI, and DCE-T1WI imaging was suggested to assess the MRI staging of EC by the European Society of genitourinary radiology in a study^23^. T2WI can clearly distinguish the structures of all layers of the uterine wall and reveal the lesions, and dynamic contrast-enhanced imaging is helpful for detecting the presence of micrangium in tissues, which is closely related to angiogenesis in tumor tissues. DWI reflects the diffusion of water molecules within tissues and is able to quantitatively reflect early pathological and physiological alterations in biological tissues. ADC can quantify the movement condition of water molecules, and researchers mostly use ADC values to predict the pathological grade, metastasis, and prognosis of a variety of tumors^24,25^. However, the ADC value can only reflect the average tumor diffusion situation, is insufficient to utilize complex signal information inside the tumor tissue, and thus cannot comprehensively detect heterogeneous informaiton of tumors. Therefore, to acquire a large amount of imaging information inside the tumor from different angles, feature extraction was performed on ADC images in this study. We extracted features from T2WI, DCE-T1WI, and ADC images, and constructed a multi-sequence joint MRI imaging radiomics model, which was compared with models constructed from a single sequence. The results showed that the AUC of the MLP model established by the joint sequences in the validation set is 0.852, and the AUC of the model from a single sequence of T2WI, DCE-T1WI, or ADC in the validation set was 0.750, 0.707, and 0.732, respectively. The model established by the joint sequences has a better predictive ability than a single sequence model. Previous studies have shown the superiority of multi-sequence models. Wang et al.^26^ investigated the MRI-based imaging omics features for predicting response to induction chemotherapy in NPC and created a separate DCE model and a combined DCE, T2WI-FS, and T2WI model, respectively. The results showed that the AUCs were 0.715 and 0.822, respectively.

In the constructed radiomics model, the two most important features were from the GLDM and NGTDM in ADC images, illustrating the advantages of ADC maps in predicting ki-67 expression levels in EC. The GLDM is the most dominant matrix in texture analysis, which reflects the statistical relationship of gray levels in neighboring pixels or voxels within a specific distance along a fixed direction. The NGTDM quantifies the sum of differences between the gray levels of a pixel or voxel and the mean gray levels of its neighboring pixels or voxels within a predefined distance. The top twenty features were mostly extracted from the T2WI map, illustrating a large amount of meaningful feature information contained in the T2WI map. It can be concluded from the SNAP plot that the larger the LDLGLE value, the more easily the ki-67 expression level is predicted. The smaller the Busyness value (Fig. 5), the easier it is to predict the ki-67 expression level.

The combined model demonstrated excellent ability to predict ki-67 expression levels in early-stage EC in fivefold cross-validation with an AUC of 0.852, worthing being used in clinical services. Calibration curves showed good fitness of the combined model, but a larger sample size is needed for further validation. DCA showed that the model combining T2WI, ADC and DCE-T1WI could provide extra profits over the “treat-all” or “treat-none” scheme when the threshold probability was 25–80%.

Current relevant machine learning models for EC are mostly constructed using the logistic regression, random forest and support vector machines^15,27,28^. In a logistic-regression radiomics model combining ADC values with texture analysis of T2WI, DWI, and DCE-T1WI sequences^20^, the preoperative ki-67 and P53 expression levels in EC patients could be successfully predicted with an AUC of 0.938 in the validation set. But the data of the study contained patients with advanced EC, which may contain different radiomics features compared with early EC. Radiomics features in advanced-stage EC are not the same as those in early-staged EC. If patients with EC of both early and advanced stages were included, the most representative features of early EC would be concealed, and the resultant radiomics model would not be able to reflect the real expression of ki-67 in early EC, thus making the model lack of clinical applicability in early-stage EC. Therefore, our study extracted multi-class radiomics features to construct a MLP machine model for predicting the ki-67 expression levels in early-stage EC. Artificial neural network (ANN) is formed by the connection of adjustable connection weights of numerous neurons, and it has the characteristics of massively parallel processing, distributed information storage and self-learning ability. Therefore, in our study, a MLP model was built to predict the ki-67 expression level of early EC. The MLP belongs to the neural network, which includes three layers: input layer, hidden layer and output layer. Different layers are connected using a full connection layer, which can be used for prediction. Ultimately, the MLP model constructed in this study had an AUC value of 0.852 for predicting the ki-67 expression in the validation set. Convolutional neural network (CNN) has been developed from MLP to be more efficient and accurate in processing image data, but requires higher sample sizes and are currently commonly used for segmentation and identification. Zhang et al.^29^ construct a CNN model for EC prediction based on features. The AUC is 0.889 in the test group with good performance, indicating that the CNN radiomics model can be used as a noninvasive marker for EC prediction. Kurata et al.^30^ segmented the uterus on MRI images using an optimized u-net architecture. It was shown to be unaffected by uterine disease when fully automated uterine segmentation was performed. For different types of tasks, choosing different architectures gives better results. In future studies, the potential of CNN for endometrial cancer-related research can be explored.

There are some limitations in this study. First, this was a single center retrospective study with a relatively small sample size, and the reproducibility and robustness of the model needed further validation. Second, manual segmentation of tumor images was applied in this study, which may affect the objectivity of segmentation results. Cases with smaller foci of early EC were discarded at the same time, resulting in a certain blind area for the model constructed in this paper. With the development of deep learning, more and more medical image automatic segmentation frameworks are proposed and are expected to be applied in the future. Finally, radiomics features were not integrated with clinical metrics and genomics. Therefore, more precision and efficient models can be constructed in the future, which will help the clinicians to achieve personalized and precision treatment of tumors early.

In conclusion, a multi-sequence MRI based radiomics model can be used to predict the ki-67 expression levels in early-stage endometrial cancer noninvasively before surgery. This provides an objective imaging basis for clinical diagnosis and treatment.

## Data Availability

The datasets generated and/or analysed during the current study are not publicly available due to the hospital policy regarding the use of datasets but are available from the corresponding author on reasonable request.
